# Imaging Adipose Tissue Browning using Mitochondrial Complex-I Tracer [^18^F]BCPP-EF

**DOI:** 10.1155/2022/6113660

**Published:** 2022-05-31

**Authors:** Julian L. Goggi, Siddesh Hartimath, Shivashankar Khanapur, Boominathan Ramasamy, Jun Rong Tang, Peter Cheng, Anna M. Barron, Hideo Tsukada, Edward G. Robins

**Affiliations:** ^1^Institute of Bioengineering and Bioimaging (IBB), Agency for Science, Technology and Research (A^∗^STAR), 11 Biopolis Way, #01-02 Helios, Singapore 138667; ^2^Neurobiology of Aging and Disease Laboratory, Lee Kong Chian School of Medicine, Nanyang Technological University Singapore, Singapore 308232; ^3^Central Research Laboratory, Hamamatsu Photonics K.K., Shizuoka 434-8605, Japan; ^4^Clinical Imaging Research Centre (CIRC), 14 Medical Drive, #B1-01, Yong Loo Lin School of Medicine, National University of Singapore, Singapore 117599

## Abstract

Browning of white adipose tissue (WAT) into beige adipocytes has been proposed as a strategy to tackle the ongoing obesity epidemic. Thermogenic stimuli have been investigated with the aim of converting existing white adipose tissue, primarily used for energy storage, into beige adipocytes capable of dissipating energy; however, evaluation is complicated by the dearth of noninvasive methodologies to quantify *de novo* beige adipocytes in WAT. Imaging with [^18^F]FDG is commonly used to measure brown adipose tissue (BAT) and beige adipocytes but the relationship between beige adipocytes, thermogenesis and [^18^F]FDG uptake is unclear. [^18^F]BCPP-EF, a tracer for mitochondrial complex-I (MC-I), acts as a marker of oxidative metabolism and may be useful for the detection of newly formed beige adipocytes. Mice received doses of the *β*3-adrenergic agonist CL-316,243 subchronically for 7 days to induce formation of beige adipocytes in inguinal white fat. PET imaging was performed longitudinally with both [^18^F]FDG (a marker of glycolysis) and [^18^F]BCPP-EF (an MC-I marker) to assess the effect of thermogenic stimulation on uptake in browning inguinal WAT and interscapular BAT. Treatment with CL-316,243 led to significant increases in both [^18^F]FDG and [^18^F]BCPP-EF in inguinal WAT. The uptake of [^18^F]BCPP-EF in inguinal WAT was significantly increased above control levels after 3 days of stimulation, whereas [^18^F]FDG only showed a significant increase after 7 days. The uptake of [^18^F]BCPP-EF in newly formed beige adipocytes was blocked by pretreatment with an adrenoceptor antagonist suggesting that beige adipocyte formation may be associated with the activation of MC-I. However, in BAT, uptake of [^18^F]BCPP-EF was unaffected by *β*3-adrenergic stimulation, potentially due to the high expression of MC-I. [^18^F]BCPP-EF can detect newly formed beige adipocytes in WAT generated after subchronic treatment with the *β*3-adrenergic agonist CL-316,243 and displays both higher inguinal WAT uptake and earlier detection than [^18^F]FDG. The MC-I tracer may be a useful tool in the evaluation of new therapeutic strategies targeting metabolic adipose tissues to tackle obesity and metabolic diseases.

## 1. Introduction

Adipose tissue is complex and plays an important role in energy homeostasis; dysfunctions can lead to increased risk of metabolic disease and obesity [1, 2]. Two functionally distinct types of adipose tissue exist, white adipose tissue (WAT) consists of white adipocytes used for energy storage (mainly fatty acids and lipids) and brown adipose tissue (BAT) consists of metabolically active, thermogenic brown adipocytes. A third type, thermogenic beige adipocytes, is inducible in WAT in response to cold, exercise or stimulation with *β*-adrenergic receptor (*β*3AR) agonists [3]. Thermogenesis in brown and beige adipocytes occurs via the action of uncoupling protein-1 (UCP1) [4]. UCP1 is located on the inner mitochondrial membrane and is associated with adaptive thermogenesis, UCP1 translocates protons through the mitochondrial intermembrane space without generating ATP, uncoupling respiration from ATP generation, resulting in energy dissipation as heat and stimulating fatty acid oxidation [5]. Adults with higher BAT mass exhibit improvements in lipid metabolism and lower body mass index, highlighting the therapeutic potential of increasing the amount or activity of BAT in overweight individuals [6, 7]. Numerous thermogenic stimuli have been investigated with the aim of converting existing WAT into beige adipocytes; however, treatment efficacy is complicated by the dearth of noninvasive methodologies to quantify de novo beige adipocyte biogenesis in WAT [8]. [^18^F]FDG is typically used in PET imaging of BAT and beige adipocytes; however, the link between beige adipocyte biogenesis and [^18^F]FDG uptake is unclear [9]. Accurate quantitation of beige adipocytes and thermogenic activity will be critical for the development of new therapeutic strategies for weight loss and lipid regulation. Beige adipocytes and brown adipocytes share similar phenotypes; both are characterized by multilocular lipid droplets and appear brown due to their abundance of mitochondria [1, 2, 10]. In contrast white adipocytes have one large lipid droplet and very few mitochondria [11, 12]. Prior studies have shown that imaging agents able to quantify mitochondrial expression can aid in the detection of new beige fat deposits in WAT after *β*3AR agonist stimulation [8, 13]. Mitochondria contain a respiratory electron transfer chain consisting of five components (complexes I-V) which are responsible for the generation of the majority of the ATP required by the cell. Mitochondrial complex I (MC-I) is the first and the largest complex in the pathway for oxidative phosphorylation [14]. In this study, we evaluated the radiopharmaceutical, 2-tert-butyl-4-chloro-5-(6-[2-(2- [18]F-fluoroethoxy)-ethoxy]pyridine-3-ylmethoxy)-2H-pyridazin-3-one (^18^F-BCPP-EF) which targets active MC-I [16] for its ability to detect the generation of beige adipocytes in inguinal WAT after subchronic *β*3AR stimulation, comparing uptake to [^18^F]FDG and confirming beige adipocyte generation using histology and immunohistochemical detection of UCP1.

## 2. Materials and Methods

### 2.1. Radiochemistry

2-(tert-butyl)-4-chloro-5-((6-(2-(2- fluoroethoxy)ethoxy)pyridin-3-yl)methoxy)pyridazin-3(2H)-one (BCPP-EF) reference standard and its tosyl-precursor were received in-kind from Hamamatsu Photonics K.K., Japan. [^18^F]BCPP-EF was prepared as previously described [14] (Supplementary Figure S1, the full radiolabeling method is described in the Supplementary Materials). The retention time of [^18^F]BCPP-EF was 7.0-7.1 min. [^18^F]BCPP-EF was isolated with a nondecay corrected radiochemical yield of 28.4 ± 4.9% within 60–63 min from aqueous [^18^F]fluoride. The radiochemical purity of [^18^F]BCPP-EF was ≥99% and molar activity (*A*_*m*_) was 32.5 ± 0.7 GBq/*µ*mol at the end of the synthesis (*n* = 3).

### 2.2. Animal Procedures

Animal procedures adhered to Institutional Animal Care and Use Committee Singapore requirements. BALB/c mice were purchased from InVivos Singapore at 6–8 weeks of age, kept in standard ABSL2 housing with food and water ad libitum. Animals (*n* = 10 per group) were dosed intraperitoneally into the left inguinal white fat pad daily for 7 days with 1 mg/kg *β*3AR agonist CL-316,243 in saline or saline alone as a control. The *β*3AR antagonist L-748,328 (1 mg/kg) was used to understand the stimulation dependency of radiotracer uptake and was administered 1 hour prior to imaging on day 7.

### 2.3. PET-CT and MR Imaging

The animals were imaged longitudinally on days 1, 3, and 7 at 60 minutes after dosing as previously described [8]. Static PET acquisitions were acquired with either [^18^F]BCPP-EF (∼10MBq per animal, 30–50 min postinjection) or [^18^F]FDG (∼5MBq per animal, 60–80 min postinjection) based on prior dynamic imaging data. Calibrated images were reconstructed and analysed using the Amide software (version 10.3 SourceForge). Uptake of radioactivity was measured using a volume of interest (VOI) around the interscapular BAT and inguinal WAT regions with the quadriceps muscle used to provide reference tissue values.

### 2.4. Histology

Inguinal fat tissues were dissected out on day 7 and fixed in 10% neutral buffered formalin, paraffin embedded and sectioned. The induction of beige adipocytes was assessed using hematoxylin and eosin (H&E) staining and immunohistochemical antibody staining for UCP1 (ab23841, Abcam). Representative images were acquired and the percentage of multilocular fat cells was assessed from the H&E stained sections and UCP1 binding was assessed from the IHC sections. Number of cells or UCP1 binding was analysed in a masked manner to treatment assignment manually, calculated as the percentage of field of view covered per 10 fields of view assessed per sample.

### 2.5. Statistical Analysis

The data were assessed for statistical significance using the GraphPad Prism version 8 (2-way ANOVA with Bonferroni post-test; *p* < 0.05 was considered statistically significant; data are expressed as mean ± SD).

## 3. Results

### 3.1. Evaluation of [^18^F]FDG and [^18^F]BCPP-EF Uptake in Browning Inguinal White Adipose Tissue

**Figure 1:**
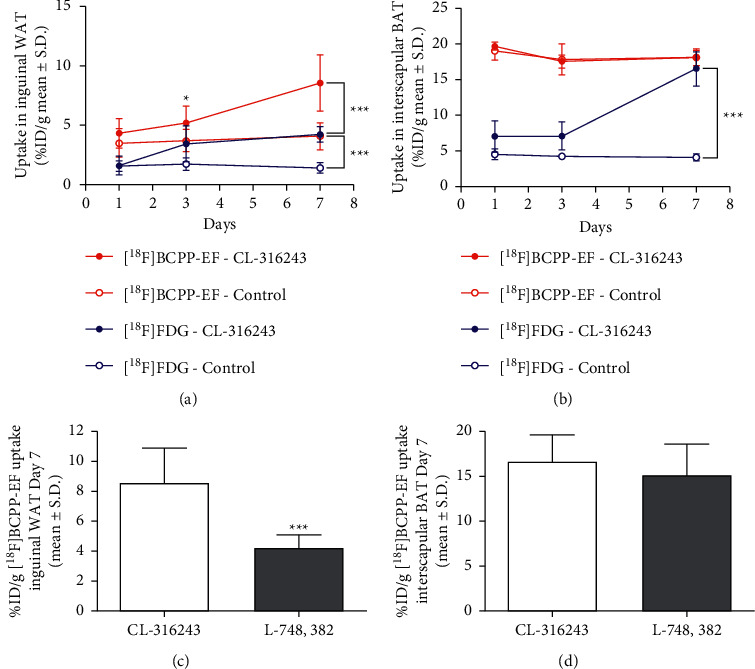
(a) Longitudinal imaging of [^18^F]BCPP-EF uptake in the inguinal fat depot treated with CL-316,243 (closed red circles) demonstrated significantly higher uptake than the control (open red circles) on days 3 and 7 post treatment (^*∗*^*p* < 0.05 and ^*∗∗∗*^*p* < 0.001). [^18^F]FDG uptake in the inguinal fat depot treated with CL-316,243 (closed blue circles) demonstrated significantly higher uptake than the control (open blue circles) on day 7 only post treatment (^*∗∗∗*^*p* < 0.001; *n* = 10 treated and *n* = 5 control; data are shown as %ID/g ± SD). (b) Longitudinal imaging of [^18^F]BCPP-EF uptake in interscapular BAT treated with CL-316,243 (closed red circles) or control (open red circles) shows no significant difference at any time point studied. Longitudinal imaging of [^18^F]FDG uptake shows significantly higher uptake in interscapular BAT treated with CL-316,243 (closed blue circles) than the control (open blue circles) at day 7 post treatment (^*∗∗∗*^*p* < 0.001; *n* = 10 treated and *n* = 5 control; data are shown as %ID/g ± SD). (c) Blockade with L-748,382 significantly reduced [^18^F]BCPP-EF uptake in inguinal WAT after subchronic treatment with CL-316,243 (^*∗∗∗*^*p* < 0.001). (d) Blockade with L-748,382 had no significant effect [^18^F]BCPP-EF uptake in interscapular BAT after subchronic treatment with CL-316,243 (*n* = 4, data are shown as %ID/g ± SD).

**Figure 2:**
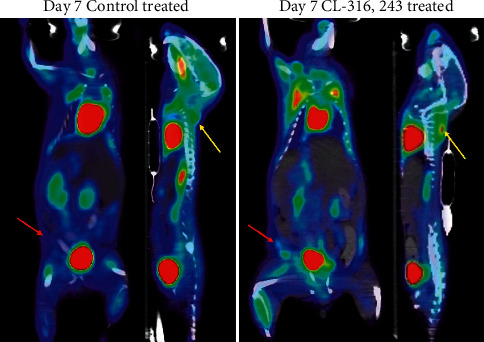
Representative image displaying the distribution of [^18^F]FDG in mice treated with the vehicle control or CL-316,243 treatment for 7 days. Inguinal white adipose depot indicated by the red arrows and interscapular BAT by the yellow arrows.

**Figure 3:**
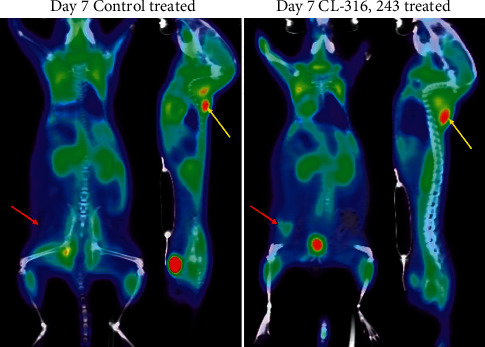
Representative image displaying the distribution of [^18^F]BCPP-EF in mice treated with the vehicle control or CL-316,243 treatment for 7 days. Inguinal white adipose depot indicated by the red arrows and interscapular BAT by the yellow arrows.

Inguinal WAT uptake of [^18^F]FDG and [^18^F]BCPP-EF was measured longitudinally in response to dosing with CL-316,243 or a saline control.  Figure 1(a) compares the change in uptake of [^18^F]FDG and [^18^F]BCPP-EF in inguinal WAT on days 1, 3, and 7 after dosing (further detail in Supplementary Tables S1 and S2). Longitudinal dosing with CL-316,243 had no significant effect on the retention of [^18^F]FDG in inguinal WAT over the first 3 days of assessment; by day 7, however, [^18^F]FDG uptake in inguinal WAT was significantly higher in the CL-316,243-treated animals than that in the control (4.23 ± 0.65%ID/g compared to 1.44 ± 0.45%ID/g, ^*∗∗∗*^*p* < 0.001, Figure 2). In contrast, the retention of [^18^F]BCPP-EF in inguinal WAT was significantly higher in CL-316,243-treated animals than that in the control after 3 days (5.20 ± 1.42%ID/g compared to 3.73 ± 0.94%ID/g, ^*∗*^*p* < 0.05) and further increased after 7 days of treatment (8.55 ± 2.36%ID/g compared to 4.08 ± 1.13%ID/g, ^*∗∗∗*^*p* < 0.001, Figure 3). This uptake of [^18^F]BCPP-EF after 7 days of treatment was significantly decreased by pretreatment with L-748,328, a *β*3AR antagonist (Figure 1(c), 4.20 ± 0.89%ID/g, ^*∗∗∗*^*p* < 0.001).

### 3.2. Evaluation of [^18^F]FDG and [^18^F]BCPP-EF Uptake in Interscapular Brown Fat

Likewise, interscapular BAT uptake of [^18^F]FDG and [^18^F]BCPP-EF was measured longitudinally in response to dosing with CL-316,243 or a saline control.  Figure 1(b) compares the change in uptake of [^18^F]FDG and [^18^F]BCPP-EF in interscapular BAT on days 1, 3, and 7 after dosing (further detail in Supplementary Tables S1 and S2). Similarly to the uptake in inguinal WAT, the uptake of FDG was only significantly increased in interscapular BAT by day 7; 16.46 ± 2.40%ID/g compared to control 4.12 ± 0.52%ID/g (^*∗∗∗*^*p* < 0.001, Figure 2). [^18^F]BCPP-EF retention, however, was unaffected by CL-316,243 treatment at any of the days studied poststimulation compared to the control (Figure 3). Pretreatment with the *β*3AR antagonist L-748,328 had no significant effect on [^18^F]BCPP-EF uptake in interscapular BAT on day 7 (Figure 1(d), 15.20 ± 3.40%ID/g).

### 3.3. Histology

**Figure 4:**
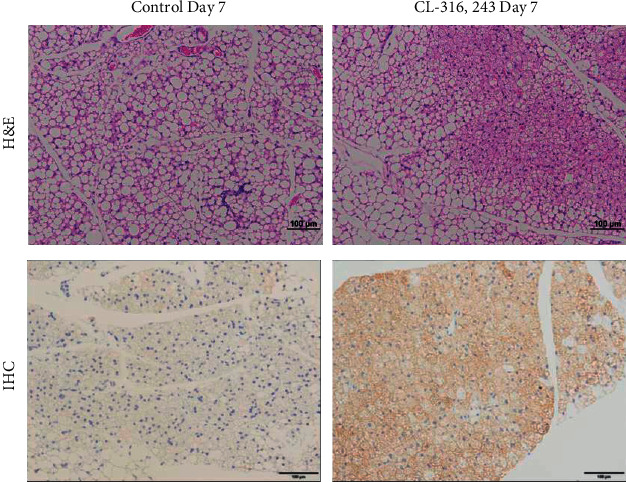
Representative inguinal WAT sections taken from CL-316,243-treated or control mice on day 7. Histological staining with H&E shows the presence of multilocular fat cells in the control or CL-316,243-treated animals (top panel), and antibody immunohistochemistry shows increased UCP1 staining in the control or CL-316,243-treated animals (bottom panel). Scale bars, 100 *µ*m.

Staining with H&E shows that the presence of multilocular fat cells has increased significantly after subchronic treatment with CL-316,243 compared to the control (25.8 ± 12.8 vs 3.6 ± 1.7, ^*∗∗*^*p* < 0.01, Figure 4 upper panel, Supplementary Figure S2A), likewise, substantial increases in UCP1 antibody staining after CL-316,243 dosing compared to the control suggestive of de novo beige adipocyte biogenesis in inguinal WAT (24.0 ± 8.1 vs 7.6 ± 3.6, ^*∗∗*^*p* < 0.01, Figure 4 lower panel, Supplementary Figure S2B).

## 4. Discussion

Many imaging techniques are able to reliably detect BAT; however, detection of beige adipocytes dispersed in existing WAT is a major challenge [17, 18]. [^18^F]FDG-PET/CT is the gold standard for imaging studies researching BAT; however, insights into the mechanism of [^18^F]FDG uptake and BAT physiology have called into question the interpretation of [^18^F]FDG uptake in thermogenic adipose tissue [9, 15]. In the current study, chemical stimulation with the *β*3AR agonist CL-316,243 caused a significant increase in inguinal beige adipocytes after 7 days of treatment (Figure 4). These activated beige adipocytes retained [^18^F]FDG as expected; however, the link between [^18^F]FDG uptake and thermogenic activity is unclear. It has been shown by indirect calorimetry that [^18^F]FDG uptake in brown adipose tissue is proportional to the amount of non‐shivering thermogenesis; however, insulin‐resistant individuals accumulate less [^18^F]FDG than their insulin-sensitive counterparts [19, 20]. Furthermore, [^18^F]FDG uptake in response to *β*3AR agonists is unaffected in UCP1 knockout mice despite defective BAT thermogenesis [21]. These findings suggest that [^18^F]FDG uptake may not be always associated with UCP1‐mediated thermogenesis. Studies estimate that glucose contributes only a small percentage of the energy required for non‐shivering thermogenesis with the majority from triglycerides [15]. In contrast, to [^18^F]FDG, which reflects glycolysis (especially anaerobic); [^18^F]BCPP-EF is considered an indicator of mitochondrial activity, reflecting oxidative metabolism [22, 23]. In non-shivering thermogenesis triglycerides are hydrolysed to fatty acids and glycerol, the resulting fatty acids undergo *β*-oxidation. As *β*-oxidation is the final step in non-shivering thermogenesis [24], [^18^F]BCPP-EF may act as a more reliable biomarker of thermogenesis. Much like [^18^F]FDG, [^18^F]BCPP-EF enabled the detection of beige adipocyte formation in inguinal WAT in response to thermogenic stimuli in an activity dependent manner. Significantly higher [^18^F]BCPP-EF uptake was observed after 3 days of stimulation, showing greater beige adipocyte uptake and earlier detection than [^18^F]FDG (Figure 1(a)). While both radiopharmaceuticals are proposed to be markers of metabolic activity, they showed different responses to *β*3AR agonist stimulation in BAT (Figure 1(b)). [^18^F]FDG uptake in BAT was increased after 7 days of treatment; however, [^18^F]BCPP-EF uptake in BAT was not, showing no significant difference from the control treated group over the time-course studied. Furthermore, while blockade of *β*3AR stimulation using the antagonist L-748,382 significantly reduced inguinal WAT uptake of [^18^F]BCPP-EF BAT uptake was unaffected (Figures 1(c) and 1(d)). The reason for the lack of [^18^F]BCPP-EF increase in BAT after *β*3AR agonist stimulation is unclear but suggests that in BAT, [^18^F]BCPP-EF may bind to MC-I independent of activity or activity dependent changes may be potentially obscured by the high number of binding sites due to the already elevated mitochondrial expression. [^18^F]BCPP-EF uptake in BAT after stimulation behaves in a similar way to the tracer [^18^F]FEPPA which binds to the 18 kDa translocator protein TSPO found in the outer leaflet of mitochondria and is a marker of mitochondrial expression as opposed to activation [8].

Overall, the data suggest that [^18^F]BCPP-EF may be a useful tool for the development of new therapeutic strategies for weight loss and lipid regulation. However, care must be taken when interpreting tracer uptake as beige adipocytes are significantly more vascularized than WAT and beige adipocyte expression is transient, after withdrawal of *β*3-AR agonist stimulation they have been observed rapidly reverting to the white phenotype [25, 26]. Further studies will be required to fully understand the relationship between [^18^F]BCPP-EF uptake and the extent of beige adipocyte formation in WAT browning in humans. Critically while *β*3AR stimulation is able to potently generate beige fat preclinically, cold acclimation and exercise regimes used to generate beige fat clinically are far less potent. Whether [^18^F]BCPP-EF is able to measure beige adipocyte activity induced by these methods remains to be seen.

## Data Availability

All the data are available on request to the corresponding author.

## Supplementary Materials

Supplementary Materials Supplementary 1: Supplementary Figure S1: radiosynthesis of [^18^F]BCPP-EF. Supplementary 2: Supplementary Figure S2: (A) figure showing % multilocular fat cells in inguinal WAT after 7 days of treatment with control or CL-316,243; (B) figure showing % UCP1 expression in inguinal WAT after 7 days of treatment with control or CL-316,243 (^*∗∗*^*p* < 0.01, ^*∗∗∗*^*p* < 0.001; *n* = 5 treated, *n* = 5 control; data are shown as %ID/g ± SD). Supplementary 3: Supplementary Table S1: table showing [^18^F]BCPP-EF uptake in the inguinal fat depot and interscapular brown adipose tissue after treatment with the control, CL-316,243, and when combined with the *β*3AR blocker L-748,382 (^*∗*^*p* < 0.05, ^*∗∗∗*^*p* < 0.001; *n* = 10 treated, *n* = 5 control, and *n* = 4 L-748,382; data are shown as %ID/g ± SD). Supplementary 4: Supplementary Table S2: table showing [18F]FDG uptake in the inguinal fat depot and interscapular brown adipose tissue after treatment with control, CL-316,243, and when combined with the *β*3AR blocker L-748,382 (^*∗∗∗*^*p* < 0.001; *n* = 10 treated, *n* = 5 control, and *n* = 4 L-748,382; data are shown as %ID/g ± SD).
