# Supplementary material for: Imaging Adipose Tissue Browning using Mitochondrial Complex-I Tracer [18F]BCPP-EF
Source: Contrast Media Mol Imaging. 2022 May 31;2022:6113660. doi: 10.1155/2022/6113660 (PMC9173993; doi:10.1155/2022/6113660)
Supplement: Supplementary Materials — Supplementary 1: Supplementary Figure S1: radiosynthesis of [18F]BCPP-EF. Supplementary 2: Supplementary Figure S2: (A) figure showing % multilocular fat cells in inguinal WAT after 7 days of treatment with control or CL-316,243; (B) figure showing % UCP1 expression in inguinal WAT after 7 days of treatment with control or CL-316,243 (∗∗p < 0.01, ∗∗∗p < 0.001; n = 5 treated, n = 5 control; data are shown as %ID/g ± SD). Supplementary 3: Supplementary Table S1: table showing [18F]BCPP-EF uptake in the inguinal fat depot and interscapular brown adipose tissue after treatment with the control, CL-316,243, and when combined with the β3AR blocker L-748,382 (∗p < 0.05, ∗∗∗p < 0.001; n = 10 treated, n = 5 control, and n = 4 L-748,382; data are shown as %ID/g ± SD). Supplementary 4: Supplementary Table S2: table showing [18F]FDG uptake in the inguinal fat depot and interscapular brown adipose tissue after treatment with control, CL-316,243, and when combined with the β3AR blocker L-748,382 (∗∗∗p < 0.001; n = 10 treated, n = 5 control, and n = 4 L-748,382; data are shown as %ID/g ± SD). [file 6113660.f1.docx]

**SUPPLEMENTARY MATERIALS**

**Imaging adipose tissue browning using the mitochondrial complex-I tracer [^18^F]BCPP-EF**

Julian L Goggi^a^*^#^; Siddesh Hartimath^a#^; Shivashankar Khanapur^a^; Boominathan Ramasamy^a^; Jun Rong Tang^a^; Peter Cheng^a^; Anna M Barron^b^ ; Hideo Tsukada^c^ and Edward G Robins^a,d^.

^a^ Institute of Bioengineering and Bioimaging (IBB), Agency for Science, Technology and Research (A*STAR), 11 Biopolis Way, #01-02 Helios, Singapore 138667.

^b^ Neurobiology of Aging and Disease Laboratory, Lee Kong Chian School of Medicine, Nanyang Technological University Singapore, Singapore, 308232.

^c^ Central Research Laboratory, Hamamatsu Photonics K.K., Shizuoka, Japan, 434-8605.

^d^ Clinical Imaging Research Centre (CIRC), 14 Medical Drive, #B1-01, Yong Loo Lin School of Medicine, National University of Singapore, Singapore 117599**.**

# Authors contributed equally

*Corresponding author:

Dr Julian L Goggi

julian_goggi@ibb.a-star.edu.sg

Tel: +65 6824 7093

1.1 General Materials

Authentic 2-(tert-butyl)-4-chloro-5-((6-(2-(2-fluoroethoxy)ethoxy)pyridin-3-yl)methoxy)pyridazin-3(2H)-one (BCPP-EF) reference standard and its’ tosyl-precursor were received in-kind from Hamamatsu Photonics K.K., Japan. Acetonitrile anhydrous (99.8 %), potassium carbonate anhydrous (99.99 %), and 4,7,13,16,21,24-hexaoxa-1,10-diazabicyclo[8.8.8]hexacosane (Kryptofix®222, 98 %) were procured from Sigma-Aldrich Pte Ltd, Singapore. HPLC-grade acetonitrile and phosphate buffered saline (PBS) 1X solution were purchased from Merck Pte. Ltd. and Thermo Fisher Scientific, respectively. All chemicals and reagents were used as supplied. [^18^F]BCPP-EF productions were carried out in a closed Thermo Scientific™ conical reacti-vial™ (5 ml). Sep-Pak® light (46 mg) accell™ plus QMA carbonate and Sep-Pak® C18 light cartridges were purchased from Waters Pacific Pte Ltd, Singapore.

No-carrier-added (nca) aqueous [^18^F]fluoride ion was produced by the irradiation of ^18^O-enriched water via the [^18^O(p,n)^18^F] nuclear reaction using a GE PETtrace 860 cyclotron. Radiochemical purification a semi-preparative HPLC system comprising of two Knaur Smartline 1050 pumps, Manual injection valve (6-port/3-channel), SmartMix 100 solvent mixer, Smartline UV-Detector 2520 (λ=254 nm) and Flow-Count radio-HPLC NaI detection system. QC analysis was performed on a UFLC Shimazdu HPLC system equipped with dual wavelength UV detector and a NaI/PMT-radiodetector (Flow-Ram, LabLogic). Radioactivity measurements were made with a CRC-55tPET dose calibrator (Capintec, USA).

1.2 Radiochemistry of [^18^F]BCPP-EF

Aqueous nca [^18^F]fluoride activity (typically, 10 GBq) was trapped on a preconditioned Sep-Pak® light (46 mg) Accell™ plus QMA carbonate (Waters). The trapped [^18^F]fluoride anion was eluted into the reaction vial using 1 ml of a 95 : 5 (v / v) acetonitrile : water mixture containing 1 mg of K_2_CO_3_ (7.2 µmol) and 5 mg of Kryptofix®222 (13.3 µmol). The [K(K_222_)]^+^[^18^F]F^-^ complex was azeotropically dried under reduced pressure and a stream of nitrogen gas (250 ml / min) at 95 °C. After the first round of drying, anhydrous acetonitrile (0.5 ml) was added to the [K(K_222_)]^+^ [^18^F]^-^ complex and the drying step was repeated. After cooling, a solution of the tosyl precursor (2.3 mg; 9.8 µmol) in anhydrous acetonitrile was added before the reaction vial was sealed and heated at 95 °C for 10 minutes (Supplementary Figure S1). After cooling to room temperature, the crude reaction mixture was diluted with 3 ml HPLC mobile phase (50 / 50 v /v  methanol / water) and purified by semi-preparative radio-HPLC using a Phenomenex Prodigy ODS-3, 5µm, 100  Å, 250 x 10 mm; 6 ml / min, λ = 254 nm). Gradient elution was carried out using a mixture of water (solvent A) and methanol (solvent B). The following gradient elution profile was used: 0.0 - 0.10 min 10 % B, 0.10 - 10.00 min 90 % B, 10.00 - 12.00 min 90 % B, 12.00-12.50 min 10% B, and 12.50 - 15.00 min 10 % B. The [^18^F]BCPP-EF pure fraction was isolated with a retention time of between 12.3-12.6 minutes. The collected HPLC pure fraction was then trapped on a pre-conditioned Sep-Pak C18 light cartridge (pre-conditioning was done by flushing the cartridge with 5 ml ethanol followed by 10 ml deionised water). The cartridge-trapped product was then washed with 5 ml of deionised water before [^18^F]BCPP-EF was eluted with 100 % ethanol (0.5 ml) and diluted with 4.5 ml of PBS. The radiochemical purity of [^18^F]BCPP-EF was assessed by analytical radio-HPLC (Agilent Zorbax Eclipse AAA, 3.5µm, 80  Å, 150 x 4.6 mm LC Column at a flow rate of 1.5 ml / min; column temperature: 40 °C, λ = 254 nm). Gradient elution was carried out using a mixture of water (solvent A) and acetonitrile (solvent B). The following gradient elution profile was used: 0.01 - 0.10 min 10 % B, 0. 10 – 0.20 min 10 % B, 0.20 - 6.00 min 95 % B, 6.00-8.00 min 95 % B, 8.00-8.30 min 10 % B, 8.30 - 12.01 min 10 % B. The retention time of [^18^F]BCPP-EF was 7.0-7.1 min. [^18^F]BCPP-EF was isolated with a non-decay corrected radiochemical yield of 28.4 ± 4.9 % within 60-63 min from aqueous [^18^F]fluoride. The radiochemical purity of [^18^F]BCPP-EF was ≥99% and molar

activity (A_m_) was 32.5 ± 0.7 GBq / µmol at the end of the synthesis (n = 3).

**Supplementary Figure S1.** Radiosynthesis of [^18^F]BCPP-EF

1.3 Histology.

Staining with H&E shows that the presence of multilocular fat cells increased significantly after sub-chronic treatment with CL-316,243 compared to control (25.8 ± 12.8 vs 3.6 ± 1.7, **p<0.01, Supplementary Figure S2A), likewise, substantial increases in UCP-1 antibody staining were observed after CL-316,243 dosing compared to control (24.0 ± 8.1 vs 7.6 ± 3.6, **p<0.01, Supplementary Figure S2B) suggestive of de novo beige adipocyte biogenesis in inguinal WAT.


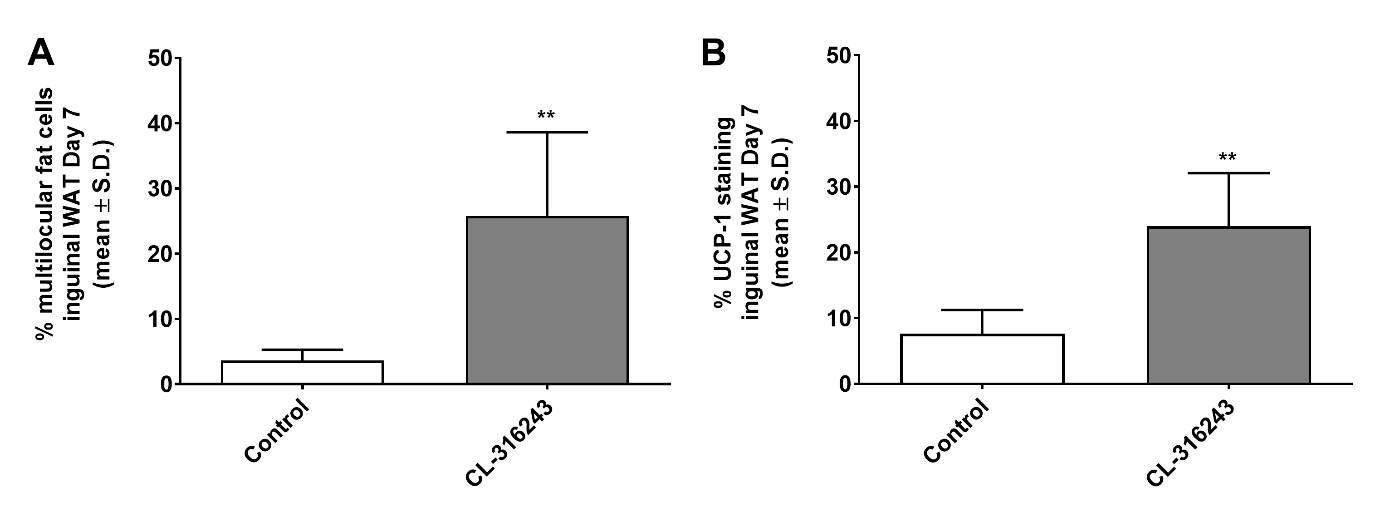


**Supplementary Figure S2.** **A.** Figure showing % multilocular fat cells in inguinal WAT after 7 days of treatment with Control or CL-316,243 **B.** Figure showing % UCP-1 expression in inguinal WAT after 7 days of treatment with Control or CL-316,243. (** p<0.01, *** p<0.001, n = 5 treated, n = 5 control, data shown as %ID/g ± SD).

| **Day** | **Inguinal WAT**  **Control**  **% ID/g [^18^F]BCPP-EF** | **Inguinal WAT**  **CL-316,243**  **% ID/g [^18^F]BCPP-EF** | **Interscapular BAT**  **Control**  **% ID/g [^18^F]BCPP-EF** | **Interscapular BAT**  **CL-316,243**  **% ID/g [^18^F]BCPP-EF** |
| --- | --- | --- | --- | --- |
| **1** | 3.52 ± 1.19 | 4.32 ± 1.26 | 18.92 ± 2.74 | 19.66 ± 1.74 |
| **3** | 3.73 ± 0.94 | 5.2 ± 1.42* | 17.78 ± 4.33 | 17.47 ± 2.88 |
| **7** | 4.08 ± 1.13 | 8.55 ± 2.36*** | 18.10 ± 2.67 | 18.01 ± 2.59 |
| **L-748,382** | 3.77 ± 0.83 | 4.2 ± 0.89*** | 18.49 ± 3.87 | 15.2 ± 3.40 |

**Supplementary Table S1.** Table showing [^18^F]BCPP-EF uptake in the inguinal fat depot and interscapular brown adipose tissue after treatment with Control, CL-316,243 and when combined with the β3AR blocker L-748,382 (* p<0.05, *** p<0.001, n=10 treated, n=5 control and n=4 L-748,382, data shown as %ID/g ± SD).

| **Day** | **Inguinal WAT**  **Control**  **% ID/g [^18^F]FDG** | **Inguinal WAT**  **CL-316,243**  **% ID/g [^18^F]FDG** | **Intercapular BAT**  **Control**  **% ID/g [^18^F]FDG** | **Interscapular BAT**  **CL-316,243**  **% ID/g [^18^F]FDG** |
| --- | --- | --- | --- | --- |
| **1** | 1.57 ± 0.45 | 1.63 ± 0.79 | 3.97 ± 0.73 | 7.00 ± 2.18 |
| **3** | 1.76 ± 0.53 | 3.45 ± 1.49 | 4.30 ± 0.14 | 7.13 ± 1.94 |
| **7** | 1.44 ± 0.45 | 4.23 ± 0.65*** | 4.33 ± 0.52 | 16.46 ± 2.40 |
| **L-748,382** | 1.49 ± 0.25 | 1.61 ± 0.37*** | 4.48 ± 0.64 | 6.41 ± 1.47 |

**Supplementary Table S2.** Table showing [^18^F]FDG uptake in the inguinal fat depot and interscapular brown adipose tissue after treatment with Control, CL-316,243 and when combined with the β3AR blocker L-748,382 (*** p<0.001, n=10 treated, n=5 control and n=4 L-748,382, data shown as %ID/g ± SD).
